# Simultaneous and systematic analysis of cellular and viral gene expression during Enterovirus 71-induced host shutoff

**DOI:** 10.1007/s13238-018-0535-6

**Published:** 2018-04-25

**Authors:** Yongquan Lin, Yan Wang, Hui Li, Yuhang Chen, Wentao Qiao, Zhi Xie, Juan Tan, Zhilong Yang

**Affiliations:** 1 Key Laboratory of Molecular Microbiology and Technology, Ministry of Education, College of Life Sciences, Nankai University, Tianjin, 300071 China; 2 Division of Biology, Kansas State University, Manhattan, KS 66506 USA; 3 State Key Laboratory of Ophthalmology, Zhongshan Ophthalmic Center, Sun Yat-sen University, Guangzhou, 500040 China

**Dear Editor,**

Enteroviruses, including poliovirus, enterovirus 71 (EV71), enterovirus 68, coxsackievirus A16, cause millions of infections every year. The infection can lead to serious human diseases and is a significant public health problem. Among them, EV71 is an emerging pathogen that causes severe hand, foot and mouth disease (HFMD) and neurological disease, especially in young children. Currently, there is no effective treatment to EV71-caused diseases, partially blaming to a lack of understanding EV71 replication mechanism (Solomon et al., 2010). As a member of *Picornaviridae*, EV71 infection induces a rapid induction of host shutoff, which is marked by the inhibition of cellular protein synthesis (Holland, 1963). In the meantime, viral protein synthesis takes over the cellular translational machinery. It is believed that the cellular protein synthesis shutoff benefits viral replication by relocating cellular resources and facilitating viral escape from host cell immune responses (Cao et al., 2017). Both transcription and translation inhibition have been suggested to contribute to the host protein synthesis shutoff during picornavirus infection (Holland, 1963; Belsham, 2009). However, the relative role of transcription and translation inhibition in virus-induced host shutoff is yet elusive. Similarly, the relative role of viral mRNA production and translation in viral protein synthesis advantage is not completely clear.

Past studies on piconavirus-induced host shutoff mainly took a reductionist approach that could not systematically and simultaneously quantitate transcription and translation. Here, we employed RNA-sequencing (RNA-Seq) and ribosome profiling, two high-throughput techniques that can simultaneously access both viral and cellular gene expression in virus-infected cells (Ingolia et al., 2011), to systematically view EV71-induced host shutoff. Total RNA from RNA-Seq indicates RNA synthesis activity, while the ribosome protected fragments (RPF) serve as the indicator of active translation. In addition, the two techniques can simultaneously access both viral and cellular gene expression in virus-infected cells. We have previously examined viral and host cell transcription and translation using these techniques in vaccinia virus infected cells (Yang et al., 2015; Dai et al., 2017). We intended to choose time points that could cover active translation of viral RNA after EV71 infection. We have determined that the active EV71 gene expression was between 3 h and 7 h post infection (hpi) using rhabdomyosarcoma (RD) cells at a multiplicity of infection (MOI) of 40 through preliminary experiments (data not shown). The use of a high MOI ensured that all cells were synchronically infected based on our preliminary experiments. Based on the results, we decided to take the snapshots of gene expression in EV71-infected and mock-infected cells at 3.5 hpi and 6.25 hpi. We carried out experiments with two independent biological replicates at each time point. In one of the experiments (Set 1), we also chose an earlier time point, 1.75 hpi, to view the early event. Total mRNA and ribosome protected RNA from EV71-infected cells and mock-infected cells were sequenced. We obtained an average of 35.3-million reads from individual RNA-Seq samples and an average of 19.4-million RPF reads from individual ribosome profiling samples that were mapped to human and EV71 genomes. The high quality of ribosome profiling reads was evidenced by high mapping rates of cellular RPF reads in coding regions (CDSs) and 5′-UTRs as well as low mapping rates of 3′-UTRs and introns for both mock and infected experiments (Fig. S1).

To evaluate the role of mRNA depletion and translation suppression in EV71-induced host shutoff, we carried out several analyses. First, the principal coordinates analysis (PCoA) showed a bigger overall change of the PRF between mock- and EV71-infected cells (Figs. 1A and S2A), suggesting a higher effect of translation regulation in this process. Second, we quantified the portions of cellular protein coding sequences in total mRNA and ribosome footprint reads. The analysis indicated much smaller portions of cellular protein coding RPF than mRNA reads at 3.5 hpi and 6.25 hpi in both sets of experiments, exemplified by 13% RPF and 47% cellular mRNA reads at 6.25 hpi in Set 2 (Figs. 1B and S2B). Finally, we plotted differentially expressed cellular genes at the mRNA and RPF levels of both sets (Figs. 1C and S2C). A higher number of downregulated cellular genes or a higher degree of downregulation was observed at the RPF levels at 3.5 hpi and 6.25 hpi. These results indicated that reduced translation of cellular mRNAs, rather than mRNA depletion, played a major role in EV71-induced host shutoff.

**Figure 1:**
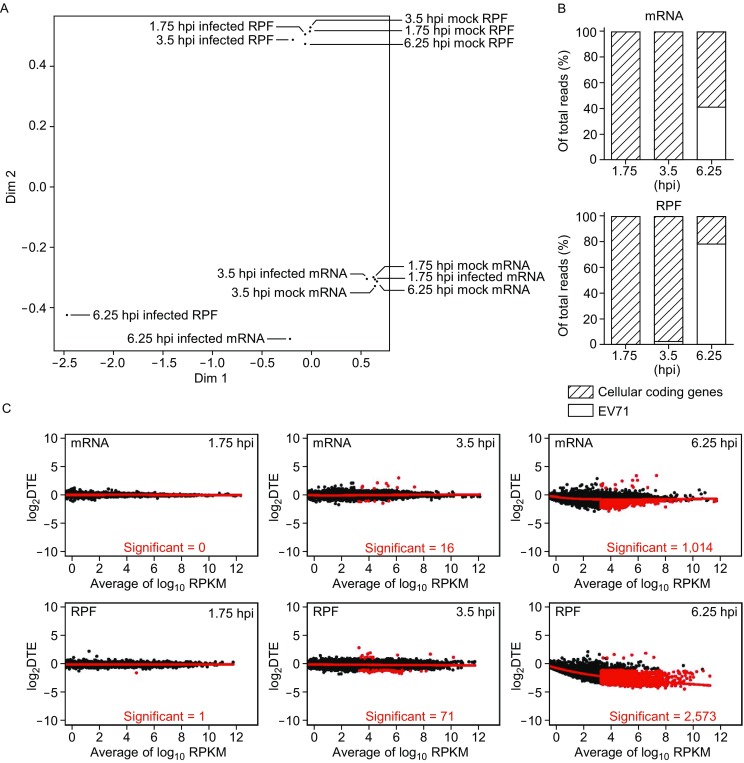
**Reduced translation of cellular mRNAs plays a key role in EV71-induced host shutoff**. (A) Principal coordinates analysis (PCoA) of gene expression shows transcriptional and translational levels of cellular gene expression (Set 1). Each point represents the indicated data point. (B) Proportion of reads mapped to EV71 RNA and cellular protein coding regions at different times after EV71 infection (Set 1). (C) Scatter plot of mean logarithmic value under mock- and EV71-infected conditions of mRNA/RPF reads versus logarithmic value of difference of mRNA/RPF (DTE) (Set 1). Genes that changed significantly (fold change >2) are shown in red. Number of up/down regulated genes are also shown

A large amount of EV71 protein is synthesized during EV71 infection. We assessed the role of viral RNA production and translation in abundant EV71 protein synthesis during EV71-induced host shutoff by analyzing the relative viral and cellular mRNA amounts and translation efficiency. Scatter plots of cellular and viral mRNA amounts against relative translation efficiency at different time points in Set 1 and Set 2 were displayed in Fig. 2A. At 3.5 hpi of both Set 1 and Set 2, when the viral RNA amounts were still low, EV71 RNA exhibited significantly higher relative translation efficiency than the majority of cellular mRNAs of both mock- and EV71-infected cells. With the progression of EV71 replication (6.25 hpi), the relative translation efficiency decreased, however, the viral RNA levels dramatically increased (Fig. 2A). The trends of the relative translation efficiency can also be viewed in box plots (Fig. S3). We also displayed EV71 mRNA and RPF reads at different times at the single-nucleotide resolution along with EV71 genomic RNA in Fig. 2B (Set 1) and Fig. S4 (Set 2). The numbers of both RNA and RPF reads increased throughout the viral genome with similar patterns during the progression of viral replication. In parallel, we plotted the ratios of RPF reads to RNA reads that reflected translation rate of the viral genomic RNA (Figs. 2B and S4). The ratio of RPF/RNA was extremely low at 1.75 hpi, suggesting low translation rate. The RPF/RNA ratio over the viral genome was the highest at 3.5 hpi, again suggesting highly active viral protein synthesis at 3.5 hpi under our experimental condition. The plot also indicated the RPF/RNA ratio became lower at 6.25 hpi (Figs. 2B and S4). Because mRNA 5′-UTR (which contains an internal ribosome entry site (IRES)) plays an essential role in translation efficiency, we further evaluated the role of EV71 5′-UTR in EV71 protein synthesis, using a luciferase reporter assay based on transfection of *in vitro* synthesized RNAs as Fig. S5 shown. We used an un-capped EV71-5UTR-firefly-luciferase-Poly(A) mRNA to mimic EV71 translation, which is cap-independent and mediated by an IRES. We used an m^7^G-capped *EIF3C*-5UTR-renilla-luciferase-Poly(A) mRNA to mimic a cellular cap-dependent translation. Firefly (EV71 5′-UTR) luciferase activities normalized by renilla luciferase (*EIF3C* 5′-UTR) were used to measure the performance of EV71 5′-UTR in mock- and EV71-infected cells. As can be seen in Figure 2C, the ratios of firefly luciferase activities increased over time, while the corresponding ratios of *EIF3C* 5′-UTR-controlled renilla luciferase activities decreased. In fact, the ratios of firefly luciferase activities normalized by renilla luciferase activities of EV71-infected to mock-infected cells increased over time, suggesting a translational advantage of EV71 5′-UTR over cellular *EIF3C* 5′-UTR (Fig. 2D). These results confirmed the translation advantage of the EV71 5′-UTR-led translation over the cellular *EIF3C* 5′-UTR-controlled translation in EV71-infected cell, caused by both the activation of cap-independent translation and suppression of cap-dependent translation.

**Figure 2:**
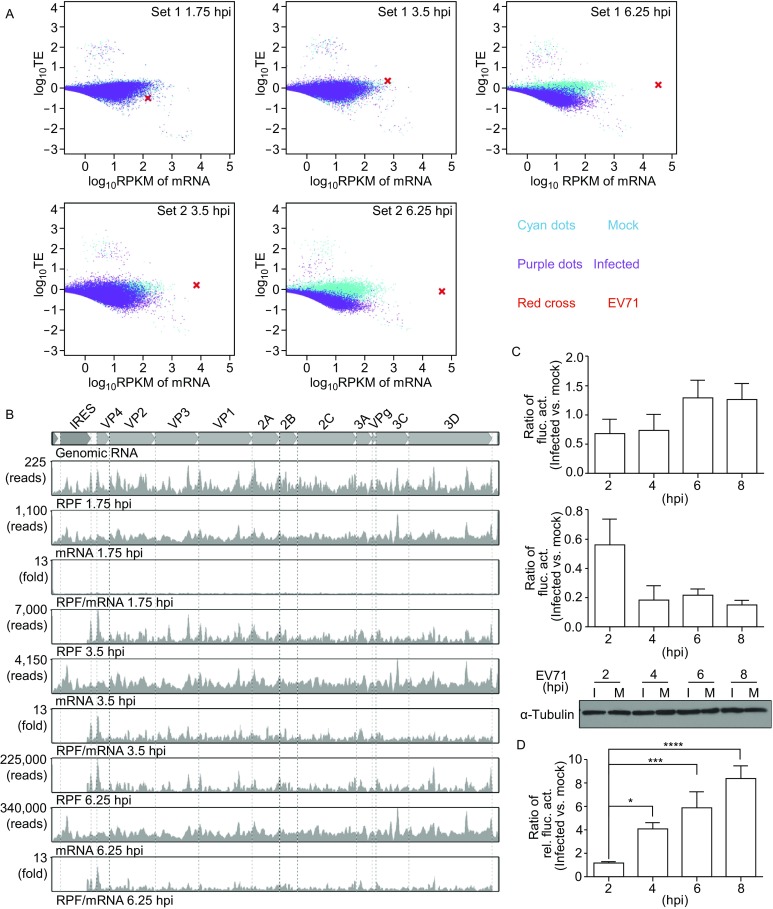
**EV71 protein is preferentially synthesized through both abundant mRNA production and advantageous mRNA translation**. (A) Log_10_TE vs. log_10_mRNA RPKM of host cell coding genes and EV71 polypeptide coding gene were plotted. Genes from mock- (cyan) and EV71-infected (purple) cells at each time point are shown in the same picture. The cross (red) shows EV71 mRNA. Only genes with a RPKM >0.3 under all conditions are shown. (B) Genome-wide EV71 RNA, RPF coverage and RPF/RNA ratio (TE). Three time points from Set 1 are shown. Data are displayed using Mochiview. EV71 genomic RNA and its subunit regions are shown on top. (C) RD cells were transfected with 480 ng un-capped EV71-5UTR-firefly-luciferase-Poly (A) mRNA and 20 ng m^7^G capped *EIF3C*-5UTR-renilla-luciferase-Poly (A) mRNAs. One hour after transfection, cells were infected with EV71 at an MOI of 40 or mock infected. At the indicated time post infection, luciferase activities were measured. The ratios of firefly luciferase and renilla luciferase activities in EV71- and mock-infected cells are shown, respectively. A Western blot analysis is shown to indicate similar amounts of cell lysates used. (D) The ratios of firefly luciferase activities normalized by renilla luciferase activities in EV71- and mock-infected cells are shown. The results are the averages of three independent experiments. The error bars indicate standard deviations. *P < 0.05, ***P < 0.001, ****P < 0.0001 (unpaired *t* test)

Many unexpected transcripts and translational products have been identified from various RNA and DNA viruses (Stern-Ginossar et al., 2012; Yang et al., 2015; Irigoyen et al., 2016). However, there is no evidence of additional translational products from either 5′-UTR, 3′-UTR, or negative sense RNA of EV71 in this study, consistent with conclusions from early studies using classical molecular approaches. In addition, it has been suggested that negative sense RNA of a virus (e.g., influenza A virus) may encode a gene (Clifford et al., 2009). Only a very few reads from negative sense EV71 RNA were associated with ribosomes, indicating no translation from EV71 negative sense RNA.

In summary, the present study for the first time simultaneously analyzed viral and host cell mRNA synthesis and translation in picornavirus-infected cells using RNA-Seq and ribosome profiling. The analyses provided a systematic overview on the dynamic change of EV71 and cellular gene expression with high resolution during EV71-induced host shutoff. Host shutoff, marked by a global cellular protein synthesis inhibition, is caused by many virus infections (Walsh and Mohr, 2011). Many of them do so by eliminating host cell mRNA pools available for translation For example, vaccinia virus encodes decapping enzymes that render cellular mRNAs sensitive to degradation and also inhibits host transcription (Parrish et al., 2007). Kaposi’s sarcoma-associated herpesvirus, and influenza A virus also deplete existing or nascent host mRNAs to block host protein synthesis (Glaunsinger and Ganem, 2004; Bercovich-Kinori et al., 2016). Our study indicates that EV71 employs a distinct approach to the aforementioned viruses mainly by a suppression of cellular mRNA translation. Because the majority of the cellular mRNAs are translated through a cap-dependent mode, while the viral mRNA is translated through an IRES-mediated cap-independent mode, the inhibition of cap-dependent translation and the activation of cap-independent translation suppressed cellular mRNA translation. Our analyses also showed that viral protein is selectively synthesized in EV71-infected cells both by employing a translational advantage and by producing a large amount of viral mRNA during EV71-induced host shutoff. At the relatively earlier time of viral infection (3.5 hpi in this study) when the viral RNA amount is not high enough, EV71 RNA is translated at a higher efficiency than most of the cellular mRNAs. At a later time of infection (6.25 hpi in this study), EV71 RNA translation efficiency decreased compared to that at 3.5 hpi. However, because of the rapid increase in viral RNA, viral proteins keep accumulating. EV71’s employment of both translational advantage and large RNA production at different stage of infection differentiates it from many other viruses. This study has broad implications in understating picornavirus-induced host shutoff. It is highly likely that other picornaviruses employ the same strategy to boost their replication. An in-depth understanding of this process following the direction pointed by this study will facilitate the development of novel anti-viral strategies by targeting EV71-induced host shutoff to reduce the public health and economic burden caused by EV71 and other enterovirus pandemic.

## Electronic supplementary material

Below is the link to the electronic supplementary material.

Supplementary material 1 (PDF 1293 kb)
