# Supplementary material for: Simultaneous and systematic analysis of cellular and viral gene expression during Enterovirus 71-induced host shutoff
Source: Protein Cell. 2018 Apr 25;10(1):72–7. doi: 10.1007/s13238-018-0535-6 (PMC6321815; doi:10.1007/s13238-018-0535-6)
Supplement: Supplementary file 1 — Supplementary material 1 (PDF 1293 kb) [file 13238_2018_535_MOESM1_ESM.pdf]

Title: Simultaneous and systematic analysis of cellular and viral gene expression during Enterovirus 71 induced host shutoff

Running title: Analysis of gene expression during EV71 induced host shutoff

Key words: Enterovirus 71, RNA-Seq, ribosome profiling, translation regulation, host shutoff

Authors and affiliations: Yongquan Lin[1, 2], Yan Wang[3], Hui Li[1], Yuhang Chen[1], Wentao Qiao[1], Zhi Xie[3]*, Juan Tan[1]*, Zhilong Yang[2]*

1 Key Laboratory of Molecular Microbiology and Technology, Ministry of Education, College of Life Sciences, Nankai University, Tianjin, 300071, China.

2 Division of Biology, Kansas State University, Manhattan, KS, 66506, USA.

3 Key Lab of Ophthalmology, Zhongshan Ophthalmic Center, Sun Yat-sen University, Guangzhou, Guangdong, 500040, China.

* Address correspondence to Zhilong Yang. Tel: +1 785 532 3602; Fax: +1 785 532 6653; Email: zyang@ksu.edu.

Or Juan Tan. Tel: +86 22 2350 3091; Fax: +86 22 2350 0590; Email: juantan@nankai.edu.cn.

Or Zhi Xie. Tel: +86 20 8733 5131; Fax: +86 20 8733 3271; Email: xiezhi@gmail.com.

Y.L. and Y.W. contributed equally to this work and are co-first authors.

SUPPLEMENTARY DATA

METHODS

Plasmid

The pGL3-basic and phRluc-TK were purchased from Promega. Plasmids used to generate PCR templates for *in vitro* RNA synthesis and reporter assays were obtained using overlapping PCR. All of the plasmids were sequenced to verify correct sequences.

Cells and virus

Human muscle rhabdomyosarcoma cell lines (RD, ATCC® CCL-136) were maintained in Dulbecco's modified Eagle's Medium supplemented with 10% fetal bovine serum (Thermo Fisher), 100 IU/ml penicillin/streptomycin, and 4 mM L-glutamine in an incubator containing 5% CO_2 at 37ºC. Human enterovirus 71 (EV71) (strain: SK-EV006/Malaysia/97) was used in this study. Genomic RNA was synthesized by *in vitro* transcription using the MEGAscript® T7 Transcription Kit (Thermo Fisher). RD cells were transfected with 10 μg RNA by Lipofectamine® 2000 (Thermo Fisher) in 10 cm dishes. Seventy-two hours post transfection, the cultured cells were frozen and thawed three times. The supernatant containing EV71 virus was harvested. To concentrate EV71 virions, the supernatant from virus-infected RD cells was ultracentrifuged at 100, 000×g for 2.5 h at 4ºC and then resuspended with an appropriate volume of DMEM. For titration, diluted virus supernatant was inoculated with RD cells in a 96-well plate. The wells with CPE were counted and the $TCID_{50}$ was calculated by the Reed-Muench method (Reed and Muench 1938).

Western blotting analysis

The supernatant from luciferase assay was mixed with 5×SDS loading buffer containing 250 mM Tris (pH 6.8), 10% SDS (w/v), 50% glycerol (v/v), 5% 2-Mercaptoethanol (v/v), and 0.5% bromphenol blue (w/v) and boiled for 10 min. Samples were resolved by SDS-PAGE and transferred onto the PVDF membrane (GE Healthcare). The membranes were blocked in 5% non-fat milk (in 1×phosphate-buffered saline) for 45 min at room temperature and probed with the indicated primary antibodies for 90 min at room temperature. After incubation with either goat anti-rabbit or goat anti-mouse secondary antibodies, the membranes were treated with the enhanced chemiluminecence reagents (Millipore), and protein signals were detected by exposure to X-ray films.

***In vitro* transcription**

RNA was synthesized using the HiScribe T7 High Yield RNA Synthesis Kit (NEB) and purified by the RNeasy Mini Kit (QIAGEN). Briefly, the DNA templates were linearized by enzyme digestion and were purified by phenol-chloroform extraction. The *in vitro* transcription reactions, including reaction buffer, ATP, UTP, CTP, GTP, T7 RNA polymerase premix, DNA template and cap analog (if needed), were set up and incubated at 37°C for 2 h.. The mRNAs were then purified using a silicon column.

Luciferase assay

RD cells were seeded at a concentration of 1.3×10^5 cells/well in a 24 well plate. The next day, the cells were transfected with 480 ng firefly luciferase mRNA and 20 ng

renilla luciferase mRNA. The amounts of the firefly and renilla mRNAs used for transfection were determined through preliminary experiments. The renilla luciferase mRNA was far less than the firefly luciferase due to its strong signal. One h after transfection, cells were infected with EV71 at an MOI of 40 or mock infected. At the indicated time post infection, the cells were collected for luciferase activity testing using the Dual-Luciferase reporter assay system (Promega).

Ribosome profiling and RNA-Seq

The TruSeq Ribo Profile (Mammalian) Kit (Illumina) was used for RNA-Seq and ribosome profiling library construction. RD cells were mock-infected or infected with EV71 at an MOI of 40. At the indicated time post infection, cells were collected for library construction following the manufacturer's instructions with minor modifications. Briefly, the cells were treated with cycloheximide (CHX) nine min before harvesting and lysis. The cell lysate was split into two portions. One portion was used for mRNA extraction using the Dynabeads® mRNA DIRECT™ Kit (Thermo Fisher), followed by RNA fragmentation. The other portion was used for ribosome protected RNA fragment purification. The RNA fragments for RNA-Seq and ribosome profiling were end repaired and ligated with adaptors. Then, reverse transcription and circularization were achieved depending on the adaptors. The rRNA and tRNA sequences were removed by annealing with biotin labeled oligos after reverse transcription. After PCR amplification by index, all the products were purified by 8% native Polyacrylamide/TBE gel (BioRad). Quality control and deep sequencing were carried out using an Illumina HiSeq 4000 platform at the Beijing Genomics

Institute (BGI).

Mapping, quantification, visualization, differential gene expression, and pathway analysis.

Briefly, adaptors of RNA-seq and ribosome profiling reads were trimmed using the FASTX Toolkit (v0.0.13.2). The tRNA and rRNA reads were removed by Bowtie (v1.0.1, -l 20)(Langmead et al. 2009). The reads were mapped to both the human genome (Ensembl, GRCh37) and EV71 genome (GenBank, AB469182.1) using Tophat (v2.0.11)(Trapnell et al. 2009). Raw counts of protein-coding genes were quantified by HTSeq (v0.6.1p2)(Anders et al. 2015). Raw counts of host genes and EV71 genes were combined, and reads per kilobase of transcript per million reads (RPKMs) were calculated by edgeR(Robinson et al. 2010). The raw read numbers were normalized to the total reads of the host cell and EV71. RPKMs of the host genes were used to calculate differentially expressed genes at both transcriptional (mRNA) and translational (RPF) levels, by comparing the expression levels in EV71-infected cells to corresponding levels in mock-infected cells at a given time point. Genes with a RPKM value ≥ 0.3 and an absolute value of fold changes ≥ 2 were identified as differentially expressed. The use of threshold of RPKM value ≥ 0.3 is to rule out those non bona fide expressed genes (Ramsköld et al. 2009). Enriched pathways were analyzed using DAVID GeneOntology(Huang et al. 2009b, a). Reads were visualized using Mochiview as instructed in the manual (Homann and Johnson 2010).

Data availability

The sequencing data were deposited at the Gene Expression Omnibus (GEO) repository. The accession number is GSE103308.

SUPPLEMENTARY REFERENCES

Anders S, Pyl PT, Huber W (2015) HTSeq--a Python framework to work with high-throughput sequencing data. Bioinforma Oxf Engl 31:166–169. doi: 10.1093/bioinformatics/btu638

Homann OR, Johnson AD (2010) MochiView: versatile software for genome browsing and DNA motif analysis. BMC Biol 8:49. doi: 10.1186/1741-7007-8-49

Huang DW, Sherman BT, Lempicki RA (2009b) Systematic and integrative analysis of large gene lists using DAVID bioinformatics resources. Nat Protoc 4:44–57. doi: 10.1038/nprot.2008.211

Huang DW, Sherman BT, Lempicki RA (2009a) Bioinformatics enrichment tools: paths toward the comprehensive functional analysis of large gene lists. Nucleic Acids Res 37:1–13. doi: 10.1093/nar/gkn923

Langmead B, Trapnell C, Pop M, Salzberg SL (2009) Ultrafast and memory-efficient alignment of short DNA sequences to the human genome. Genome Biol 10:R25. doi: 10.1186/gb-2009-10-3-r25

Ramsköld D, Wang ET, Burge CB, Sandberg R (2009) An abundance of ubiquitously expressed genes revealed by tissue transcriptome sequence data. PLoS Comput Biol 5:e1000598. doi: 10.1371/journal.pcbi.1000598

Reed LJ, Muench H (1938) A Simple Method of Estimating Fifty Per Cent Endpoints,. Am J Epidemiol 27:493–497

Robinson MD, McCarthy DJ, Smyth GK (2010) edgeR: a Bioconductor package for differential expression analysis of digital gene expression data. Bioinforma Oxf Engl 26:139–140. doi: 10.1093/bioinformatics/btp616

Trapnell C, Pachter L, Salzberg SL (2009) TopHat: discovering splice junctions with RNA-Seq. Bioinforma Oxf Engl 25:1105–1111. doi: 10.1093/bioinformatics/btp120

SUPPLEMENTARY FIGURES AND FIGURE LEGENDS

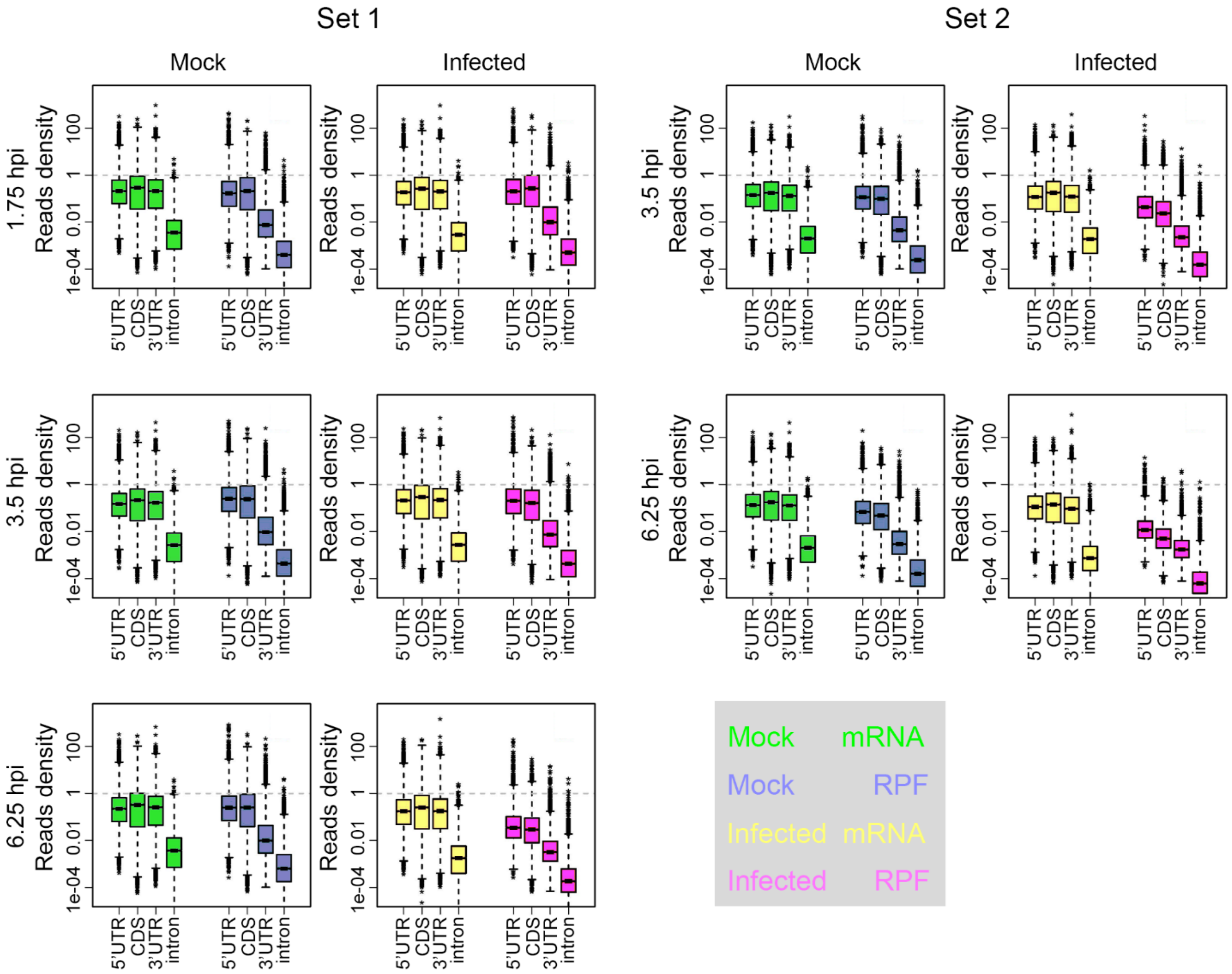

SUPPLEMENTARY FIGURE 1. Read densities of cellular mRNAs on genomic regions. Reads densities on cellular mRNA 5'UTR, CDS, 3'UTR and intron are shown.

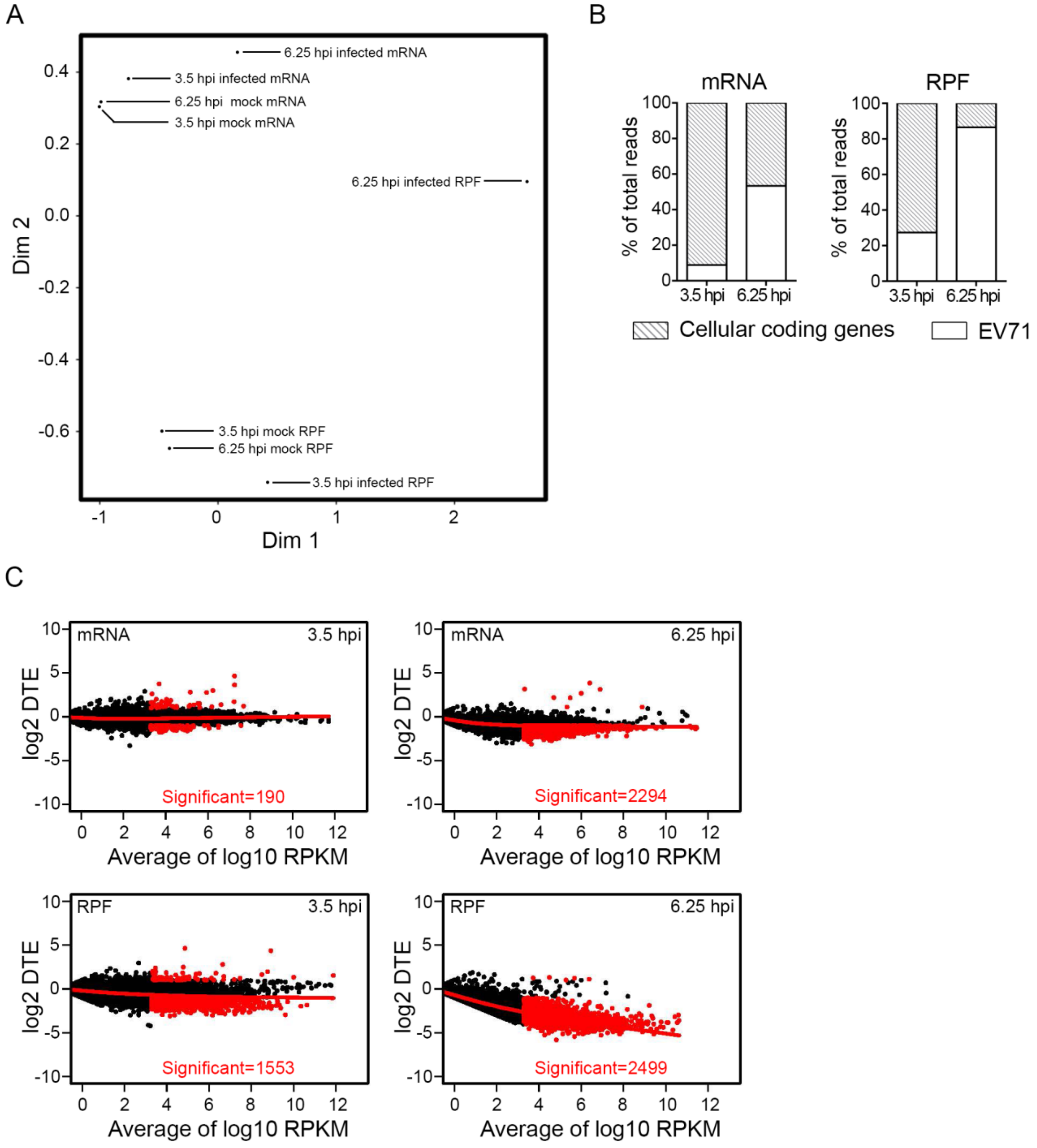

SUPPLEMENTARY FIGURE 2. Reduced translation of cellular mRNAs plays a key role in EV71-induced host shutoff. (A) Principal coordinates analysis (PCoA) of gene expression shows transcriptional and translational levels of cellular gene expression (Set2). Each point represents the indicated data point. (B) Proportion of reads mapped to EV71 RNA and cellular protein coding regions at different times

after EV71 infection (Set2). (C) Scatter plot of mean logarithmic value under mock- and EV71-infected conditions of mRNA/RPF reads versus logarithmic value of difference of mRNA/RPF (DTE) (Set2). Genes that changed significantly (fold change >2) are shown in red. Numbers of up/down regulated genes are also shown.

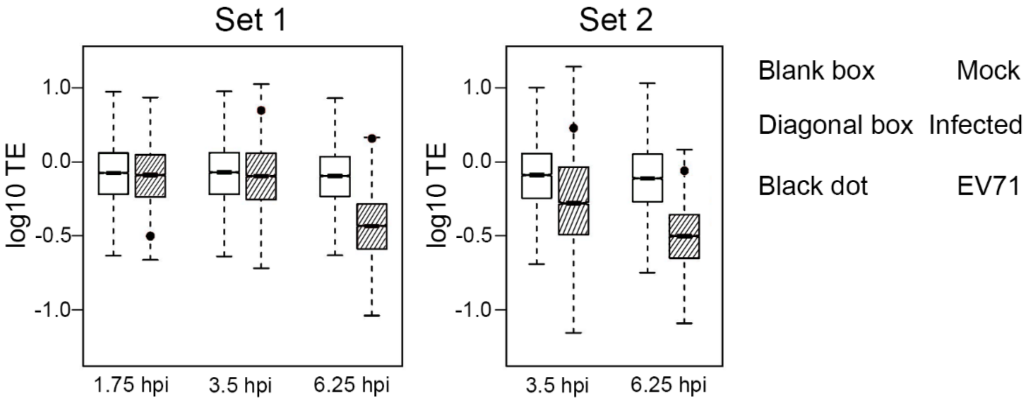

SUPPLEMENTARY FIGURE 3. Box plots of relative translation efficiency. Box plot of host cell coding genes and EV71 polypeptide coding gene of mock (blank) and infected (diagonal). Black dot represents EV71 mRNA. Only genes have a RPKM>0.3 at all condition are calculated.

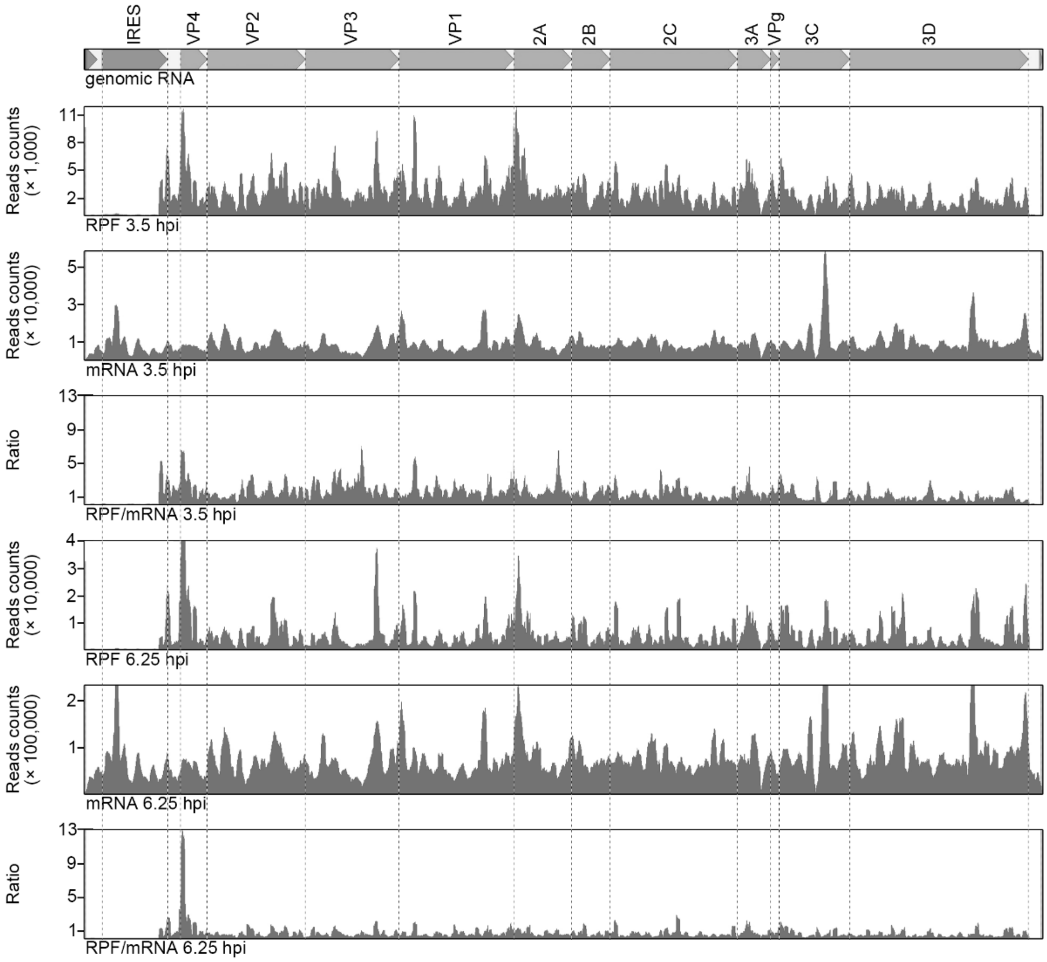

SUPPLEMENTARY FIGURE 4. Genome-wide EV71 RNA, RPF coverage, and RPF/RNA ratio (TE). Two time points from Set 2 are shown. Data are displayed using Mochiview. EV71 genomic RNA and its subunit regions are shown on top.

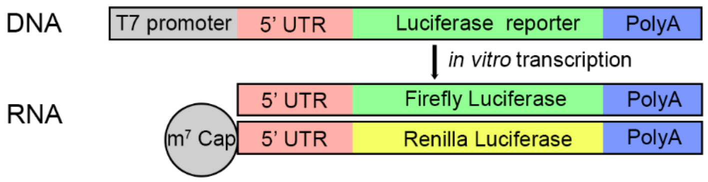

SUPPLEMENTARY FIGURE 5. Schematic diagram of the DNA template and in vitro transcribed RNA used in the reporter assay. RNA was *in vitro* synthesized using T7 polymerase with a DNA template containing a T7 promoter, a desired 5' UTR sequence (from EV71 genome or *EIF3C* mRNA), a luciferase ORF (firefly or renilla luciferase), and a poly(A) tail-encoding sequence. A 5' end m^7G cap was also added to the RNA with *EIF3C* 5' UTR.
